# Identification and Antimicrobial Resistance of *Enterococcus* Spp. Isolated from the River and Coastal Waters in Northern Iran

**DOI:** 10.1155/2014/287458

**Published:** 2014-11-26

**Authors:** Majid Alipour, Reza Hajiesmaili, Maryam Talebjannat, Yousef Yahyapour

**Affiliations:** ^1^Department of Microbiology, Babol Branch, Islamic Azad University, Babol, Iran; ^2^Department of Microbiology, Infectious Diseases & Tropical Medicine Research Center, Faculty of Medicine, Babol University of Medical Sciences, Babol, Iran

## Abstract

As fecal streptococci commonly inhabit the intestinal tract of humans and warm blooded animals, and daily detection of all pathogenic bacteria in coastal water is not practical, thus these bacteria are used to detect the fecal contamination of water. The present study examined the presence and the antibiotic resistance patterns of *Enterococcus* spp. isolated from the Babolrud River in Babol and coastal waters in Babolsar. Seventy samples of water were collected in various regions of the Babolrud and coastal waters. Isolated bacteria were identified to the species level using standard biochemical tests and PCR technique. In total, 70 *Enterococcus* spp. were isolated from the Babolrud River and coastal waters of Babolsar. *Enterococcus faecalis* (68.6%) and *Enterococcus faecium* (20%) were the most prevalent species. Resistance to chloramphenicol, ciprofloxacin, and tetracyclin was prevalent. The presence of resistant *Enterococcus* spp. in coastal waters may transmit resistant genes to other bacteria; therefore, swimming in such environments is not suitable.

## 1. Introduction

The entrance of rural, urban, and industrial wastewaters into the rivers and seas with their high level of pathogenic and other polluting agents threatens public health. One of the routes of transmission for marine borne disease is direct exposure to beach environments. Direct exposure includes accidental ingestion of contaminated water or exposing skin, eyes, and ears to contaminated waters during swimming [1]. Several studies have found that bathing in recreational waters fecally contaminated has been associated with an increased risk of transmission of infectious diseases including gastroenteritis and respiratory, skin, eyes, and ears illnesses [2]. Enterococci are members of the intestinal microbiota of healthy humans and animals and can be released into the environmental sources such as soil and surface water by human and animal fecal material [3, 4]. Some enterococcal strains have also been used as human probiotics because they can survive and compete in the gastrointestinal tract [5]. There are currently 43 species in this genus; however, relatively few species are important human pathogens. The most commonly isolated and clinically important species are* Enterococcus faecalis* and* Enterococcus faecium*. Other species such as* Enterococcus gallinarum* and* Enterococcus casseliflavus* are rarely pathogenic in humans [6]. The enterococci grow both aerobically and anaerobically in a broad temperature range (10°C to 45°C), in a wide pH range (4.6 to 9.9), and in the presence of high concentrations of sodium chloride (NaCl) and bile salts [7]. Enterococci may survive longer in marine environments by their capacity to tolerate high salts concentrations [8]. The spectrum of infections caused by the enterococci includes urinary tract infections (UTIs), wound infections, and bacteremia. They are also frequently associated with endocarditis, intra-abdominal, and pelvic infections [9]. Enterococci are commonly used as fecal indicators in the aquatic environment because of their abundance in feces and their long survival in the environment [10].* E*.* faecalis* and* E*.* faecium* are important enterococcal species used as fecal pollution indicators, because they are the main enterococci derived from human intestine and feces [11]. The objectives of this study were to describe the species distribution and to evaluate the antimicrobial resistance of bacteria from the genus* Enterococcus*, which were isolated from the river and coastal waters.

## 2. Materials and Methods

This research was carried out in the Babolrud River of Babol and coastal waters of Babolsar (Mazandaran province, Northern Iran). Samples were collected from June 2013 to Jan 2014. The Babolrud River originates from Savadkooh Mountains, then flows through Babol, and finally unites with coastal waters of Babolsar. Water samples were taken from various regions of the Babolrud and coastal waters of Babolsar.

### 2.1. Detection of Fecal Enterococci

Sterile glass bottles (500 mL) were used to collect water samples in duplicate. Water samples were collected at a depth of 30 cm by aseptic techniques. The samples were placed in a sealed container with dry ice and transported to the laboratory. In the laboratory, samples were refrigerated immediately. The microbiological analyses were performed within 24 h of sampling. Seventy samples of water were collected in various regions of the Babolrud and coastal waters, during different hours. For detection of fecal enterococci, 100 mL of water was filtered onto 0.45 *μ*M membrane filters (Millipore) and filter membranes were incubated onto Pfizer selective enterococcus (PSE) agar 37°C for 48 h. After the incubation, black colonies surrounded by the characteristic dark brown to black zones were taken as presumptive enterococci.

### 2.2. Bacterial Identification

Enterococci were identified to the genus level by Gram staining, catalase test, and growth in NaCl 6.5% broth, as well as growth and esculin hydrolysis on bile-esculin agar [12].

### 2.3. Confirmation of* Enterococcus* spp. by Polymerase Chain Reaction (PCR)

Species identification (*Enterococcus faecalis*,* E. faecium*,* E. gallinarum*, and* E. casseliflavus*) was performed by PCR as previously described [13]. The specific primer sets used in manganese-dependent superoxide dismutase (*sodA*) genes based PCR analysis for the identification of* Enterococcus* spp. are shown in Table 1.

The DNA was isolated from colonies by suspending the colonies in 50 *μ*L of deionized water and boiled for 15–20 min to liberate the nucleic acid [13]. Two PCR master mixes consisting of different primer sets were prepared. Group 1 was* E. faecalis* and* E. faecium*; group 2 was* E. casseliflavus* and* E. gallinarum*. The reaction mixtures (final volume, 25 *μ*L) contained 3 *μ*L of the solution containing DNA, 2.5 *μ*L of 10x reaction buffer, 1.5 *μ*L of 25 mM MgCl_2_, 1 *μ*L of Taq polymerase (5 U/*μ*L), 5 *μ*L of deoxynucleoside triphosphates (1 Mmol), 1 *μ*L of each primer (20 pmol), and 10 *μ*L of distilled water. The reactions were performed as follows: initial denaturation at 94°C for 3 min, followed by 35 cycles of denaturation at 94°C for 1 min, annealing at 38°C (for group 1) and 45°C (for group 2) 1 min, extension at 72°C for 1 min, and a final extension at 72°C for 7 min. Positive and negative DNA controls were included in all assays. The amplified products were separated by electrophoresis in ethidium bromide-stained 2% agarose gels in Tris-borate-EDTA buffer at 120 V for 30 min. The gels were visualized with a UV transilluminator.

### 2.4. Antibiotic Susceptibility Testing

All enterococci isolates were tested for resistance to six antibiotics. Susceptibility to vancomycin (30 *μ*g), ampicillin (10 *μ*g), tetracycline (30 *μ*g), ciprofloxacin (5 *μ*g), chloramphenicol (30 *μ*g), and gentamicin (10 *μ*g) was determined by disk diffusion method according to the Clinical and Laboratory Standards Institute guidelines [14]. Diameters of zones of inhibition were recorded in millimeters and interpreted as sensitive or resistant. Organisms considered intermediate by this method were recorded as resistant. Reference strain* Enterococcus faecalis* ATCC 29212 was used as control strain.

### 2.5. Statistical Analysis

Statistical Analysis was performed using SPSS software. The chi-square test was used to test the statistical differences.

## 3. Results

The enterococci were detected in all analyzed water samples.

**Figure 1:**
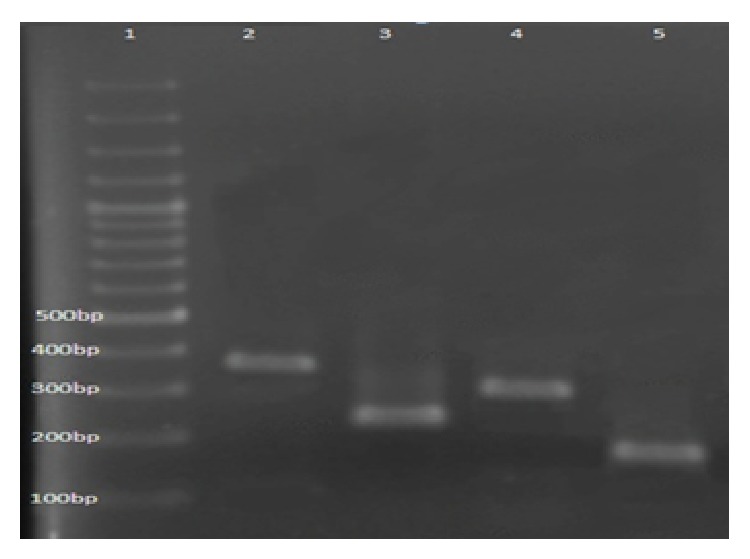
PCR products amplified with different primers and separated on agarose gel electrophoresis. Lane 1, molecular weight ladder; Lane 2,* E. faecalis*; Lane 3,* E. faecium*; Lane 4,* E. casseliflavus*; Lane 5,* E. gallinarum*.

In total, 70 enterococci were isolated from the Babolrud River and coastal waters of Babolsar. Of these, 40 were isolated from coastal water and 30 the Babolrud River. Four different* Enterococcus* spp. were confirmed by PCR:* E. faecalis*,* E. faecium*,* E. gallinarum,* and* E. casseliflavus* (Figure 1).

The species distribution of isolates was as follows:* Enterococcus faecalis* (68.6%),* Enterococcus faecium* (20%),* Enterococcus gallinarum* (7.1%), and* Enterococcus casseliflavus* (4.3%) (Table 2).

The most frequently isolated* Enterococcus* species were* E. faecium* and* E. faecalis*. The pattern of antimicrobial resistance of strains is shown (Table 3).

Antimicrobial resistant patterns were determined for 48* E. faecalis*, 14* E. faecium*, 5* E. gallinarum,* and 3* E. casseliflavus* isolated. The percentage of* Enterococcus* spp. isolated exhibited resistance to ampicillin (20%), vancomycin (4.2%), tetracycline (28.6%), gentamycin (15.7%), chloramphenicol (34.3%), and ciprofloxacin (30%). Resistance was highest to chloramphenicol (34.3%) and least to vancomycin (4.2%).* E. faeccium* showed the least resistance to vancomycin and gentamycin (7.1%).

## 4. Discussion

The distribution of enterococci, especially* E. faecalis* and* E. faecium,* in environmental water poses a potential risk to human health and should be monitored closely [15]. The presence of enterococci is considered as an indicator of fecal contamination of environmental water sources [16]. Enterococci have some advantages over coliform bacteria as indicators of hygienic safety of water resources. They are more resistant to adverse environmental conditions [17]. Enterococci are associated with health risk to swimmers [18]. The present study showed the presence of enterococci in all of the river and coastal water samples analyzed. In our study, four* Enterococcus* species including* E. faecalis*,* E. faecium*,* E. gallinarum,* and* E. casseliflavus* were identified.* E*.* faecalis* is the most frequent species isolated from the River and coastal bathing water samples (68.6%) and this is in agreement with reports of de Oliveira and Pinhata and Pinto et al. [19, 20]. The results obtained in this study indicated that* E. faecium* was one of the major* Enterococcus* species in water samples (20%). These findings indicated that there was a source of fecal contamination in the river and coastal bathing waters. These contaminations are caused by different factors, including wastes from domestic and workshops and so forth, which are located by the river. In addition, sewage from hotels and houses is dumped into the coastal bathing water of Babolsar. Wade et al. reported a strong relationship between enterococci and illness among swimmers in coastal waters [21]. 20% of the* Enterococcus* spp. isolated in current study was resistant to ampicillin, while in Kimiran-Erdem et al. study 6% of strains isolated were resistant to ampicillin [22]. In another study, Rathnayake et al. reported that 27.3% of water sourced strains of all the* E. faecium* isolates were resistant to ampicillin [15]. It appears that the results of these studies may be associated with the usage of the antibiotic in different areas. Łuczkiewicz et al. [23] reported 22% of* Enterococcus* spp. isolated from surface water, resistant to ciprofloxacin but in the present study 30% of isolates exhibited resistance to ciprofloxacin. Fluorinated quinolones were used frequently in this region, so a high percentage of enterococci exhibited resistance to ciprofloxacin. The level of vancomycin resistance (4.2%) observed in this study was in concert with the study conducted on* Enterococcus* spp. isolated (3.8%) from recreational waters [19]. Our results showed that the* Enterococcus* spp. isolates were sensitive to vancomycin, probably because this antibiotic has not been highly used in this region to date. Moore et al. [24] reported that a high percentage of* E. faecium* and* E. faecalis* isolates exhibited resistance to tetracycline which was consistent with our results. Kimiran-Erdem et al. [22] reported that 3% of the seawater samples contained resistance to chloramphenicol but in this study chloramphenicol resistance was 15.7%. The reason for this difference can be related to use of chloramphenicol in the area. In conclusion, the results of the study showed the presence of antibiotic resistant* Enterococcus* spp. in the river and coastal waters. The presence of* Enterococcus* spp. resistant to various types of antibiotics in coastal waters may cause illness among swimmers.

## Figures and Tables

**Table 1:** PCR primers used in this study.

Species	Primers	Sequence (5′-3′)	Product size (bp) reference
*E. faecalis *	F	ACT TAT GTG ACT AAC TTA ACC	360
	R	TAA TGG TGA ATC TTG GTT TGG	

*E. faecium *	F	GAA AAA ACA ATA GAA GAATTAT	215
	R	TGC TTT TTTGAA TTC TTC TTT A	

*E. gallinarum *	F	TTA CTT GCT GAT TTT GAT TCG	173
	R	TGA ATT CTT CTT TGA AAT CAG	

*E. casseliflavus *	F	TCC TGA ATT AGG TGA AAA AAC	288
	R	GCT AGT TTA CCG TCT TTA ACG	

**Table 2:** Species distribution of *Enterococcus* spp. isolated.

Source	Number of isolates	Number (%) of isolates			
		*E. faecalis *	*E. faecium *	*E. gallinarum *	*E. casseliflavus *
Babolrud River	30	20 (66.7)	7 (23.3)	2 (6.6)	1 (3.3)
Coastal water	40	28 (70)	7 (17.5)	3 (7.5)	2 (5)

Total	70	48 (68.6)	14 (20)	5 (7.1)	3 (4.3)

**Table 3:** Antibiotic resistance patterns among *Enterococcus* spp. isolated from waters.

Species	Number of isolates (%)	Number (%) of isolates					
		AP	VA	TET	GM	C	CP
*E. faecalis *	48 (68.6)	8 (16.6)	2 (4.1)	14 (29.1)	10 (21)	20 (41.6)	15 (31.2)
*E. faecium *	14 (20)	5 (35.7)	1 (7.1)	5 (35.7)	1 (7.1)	4 (28.5)	5 (35.7)
*E. gallinarum *	5 (7.1)	1 (20)	1 (20)	—	—	1 (20)	—
*E. casseliflavus *	3 (4.3)	—	—	—	—	—	—

Total	70 (100)	14 (20)	3 (4.2)	20 (28.6)	11 (15.7)	24 (34.3)	21 (30)

AP, ampicillin; VA, vancomycin; TET, tetracycline; GM, gentamycin; C, chloramphenicol; CP, ciprofloxacin.
